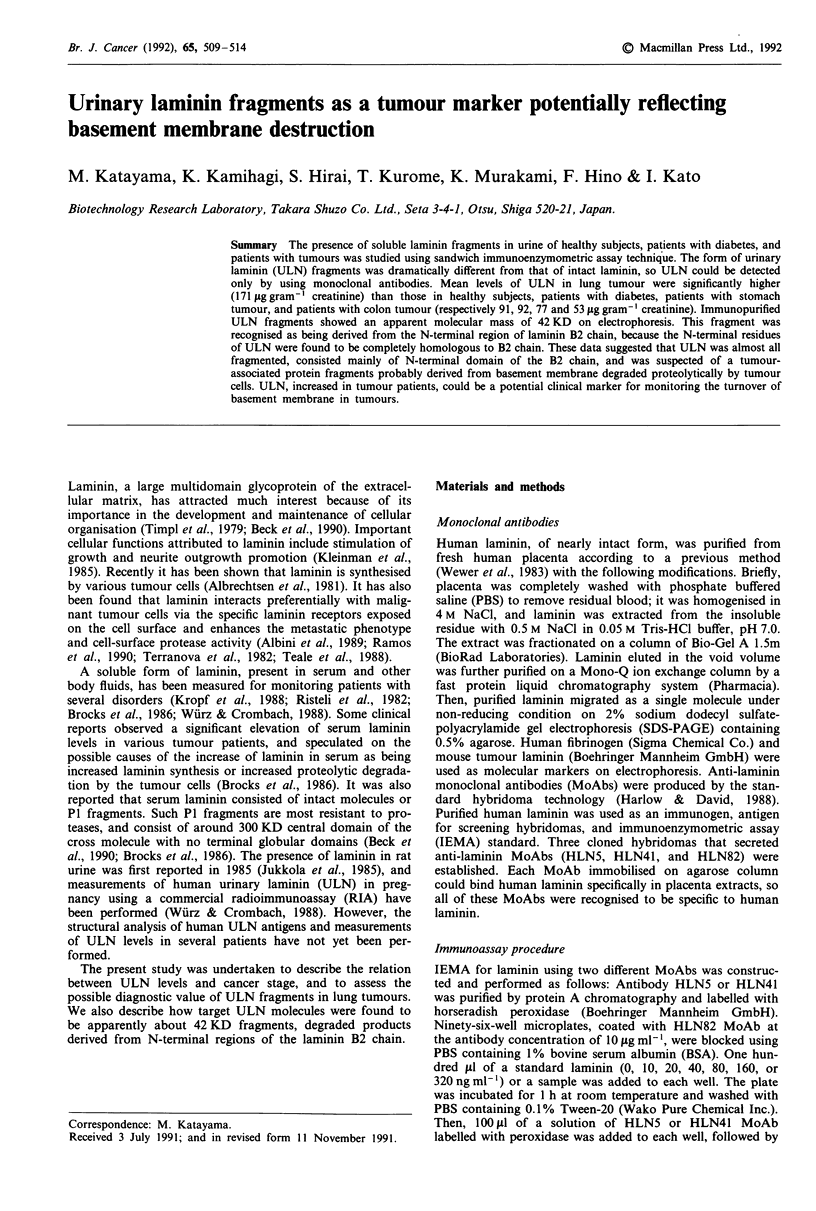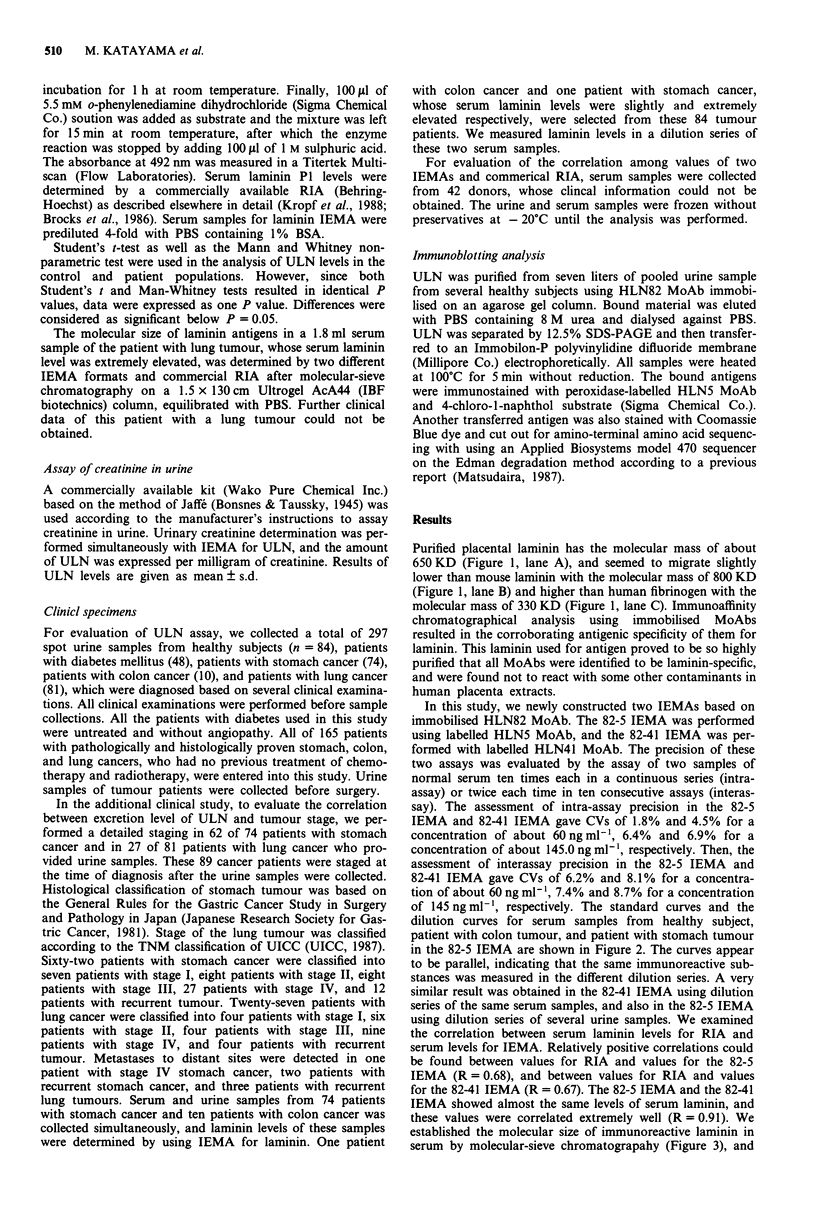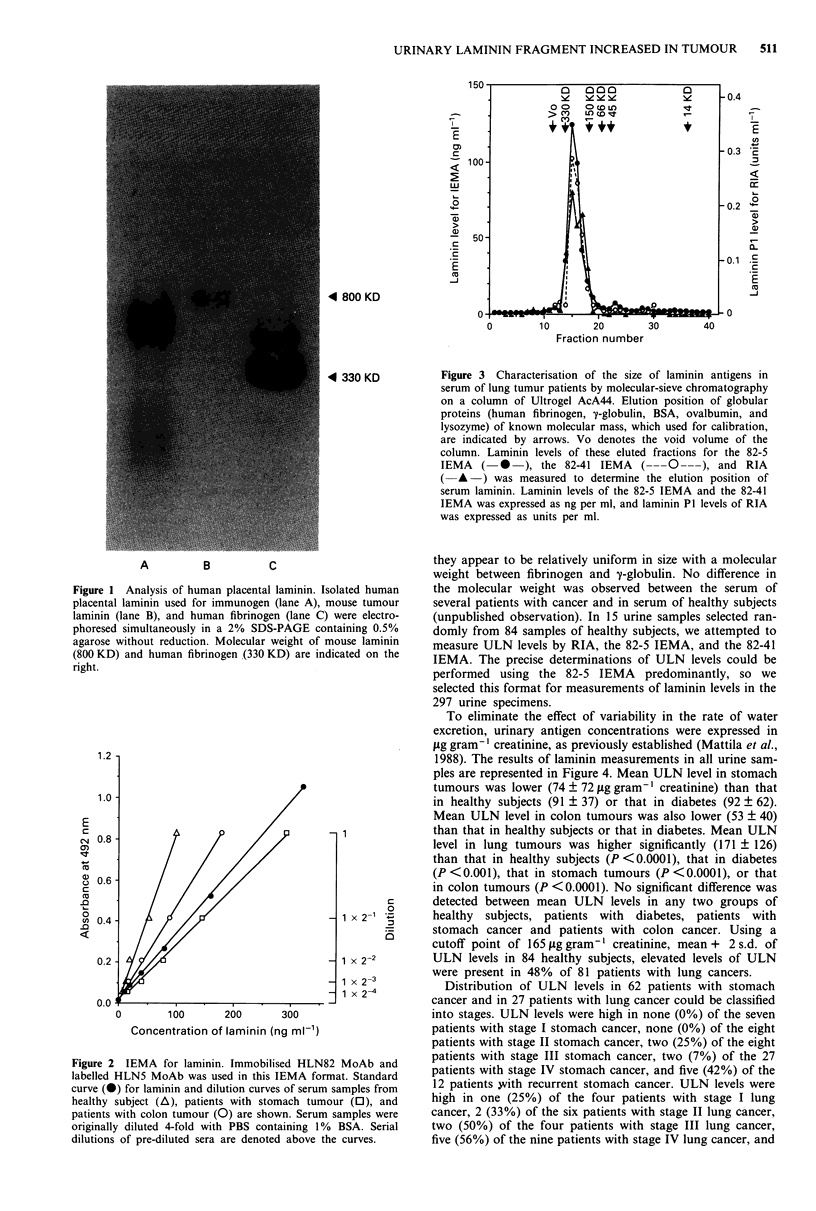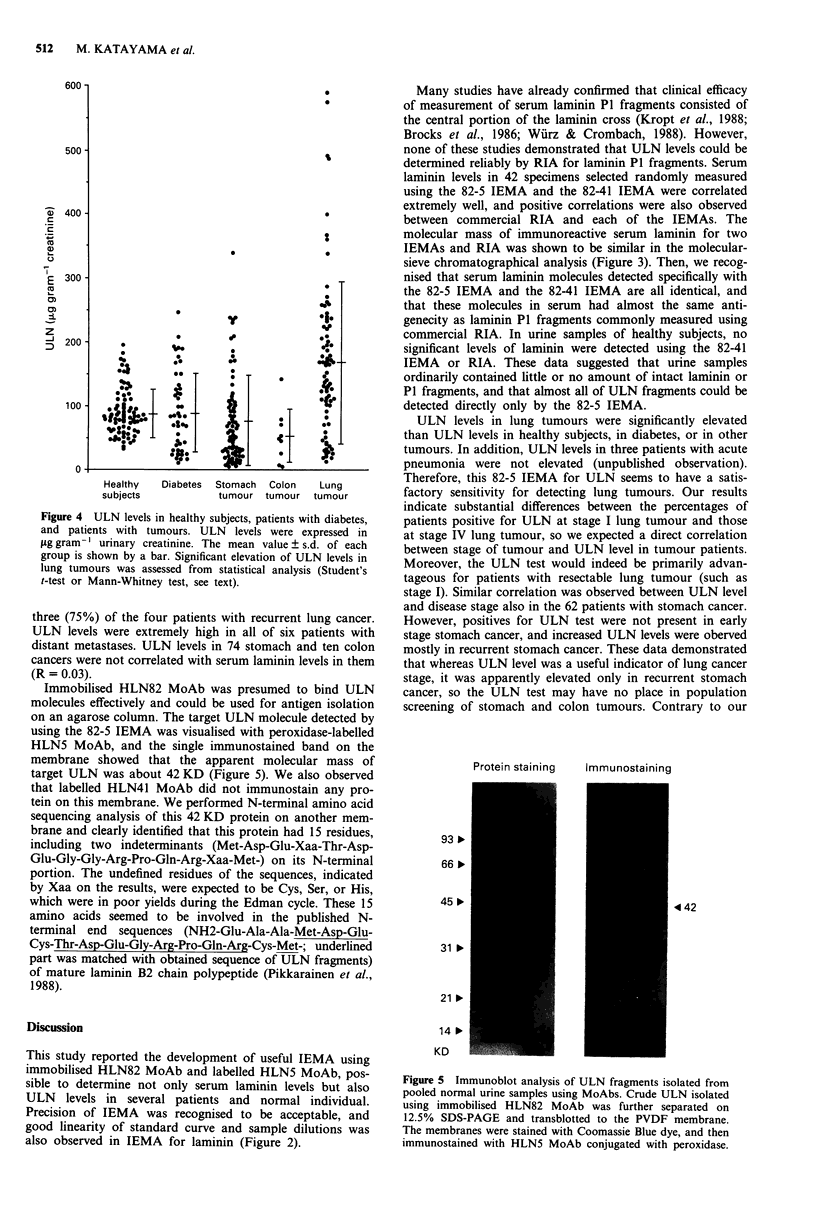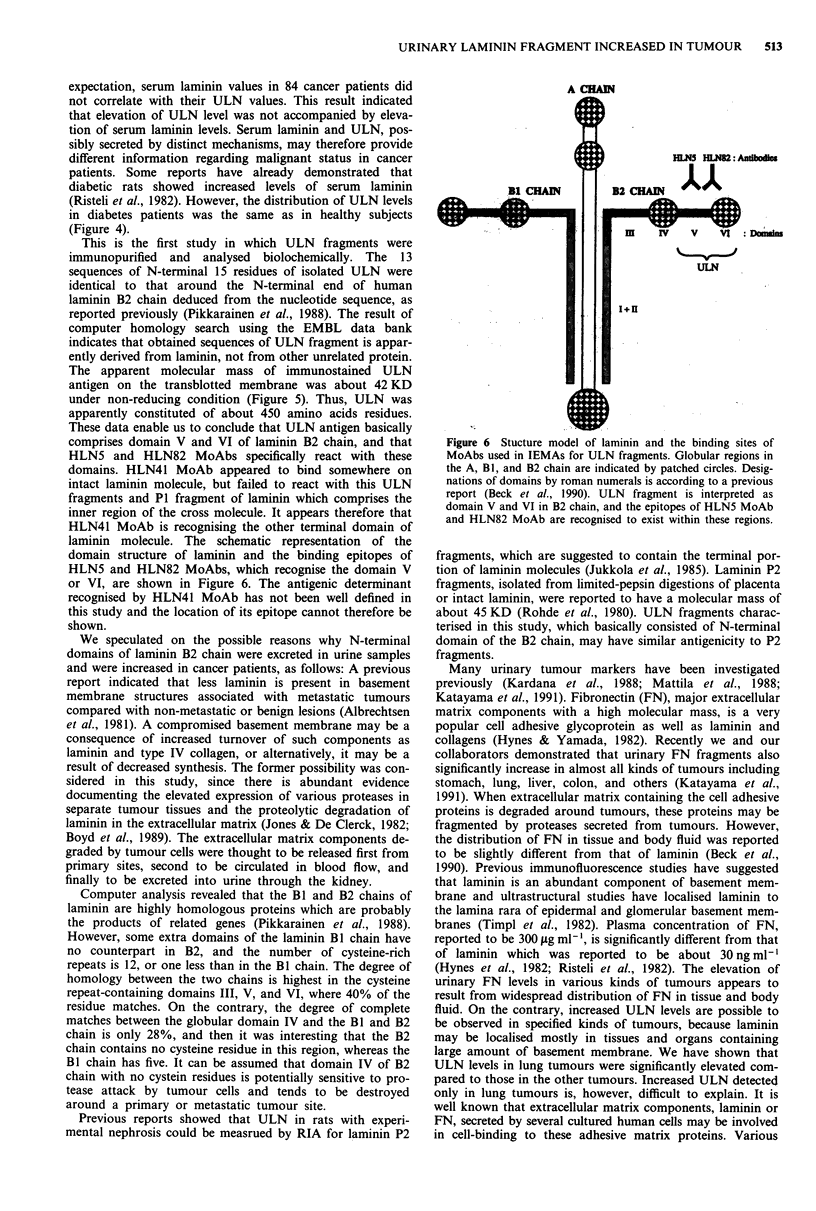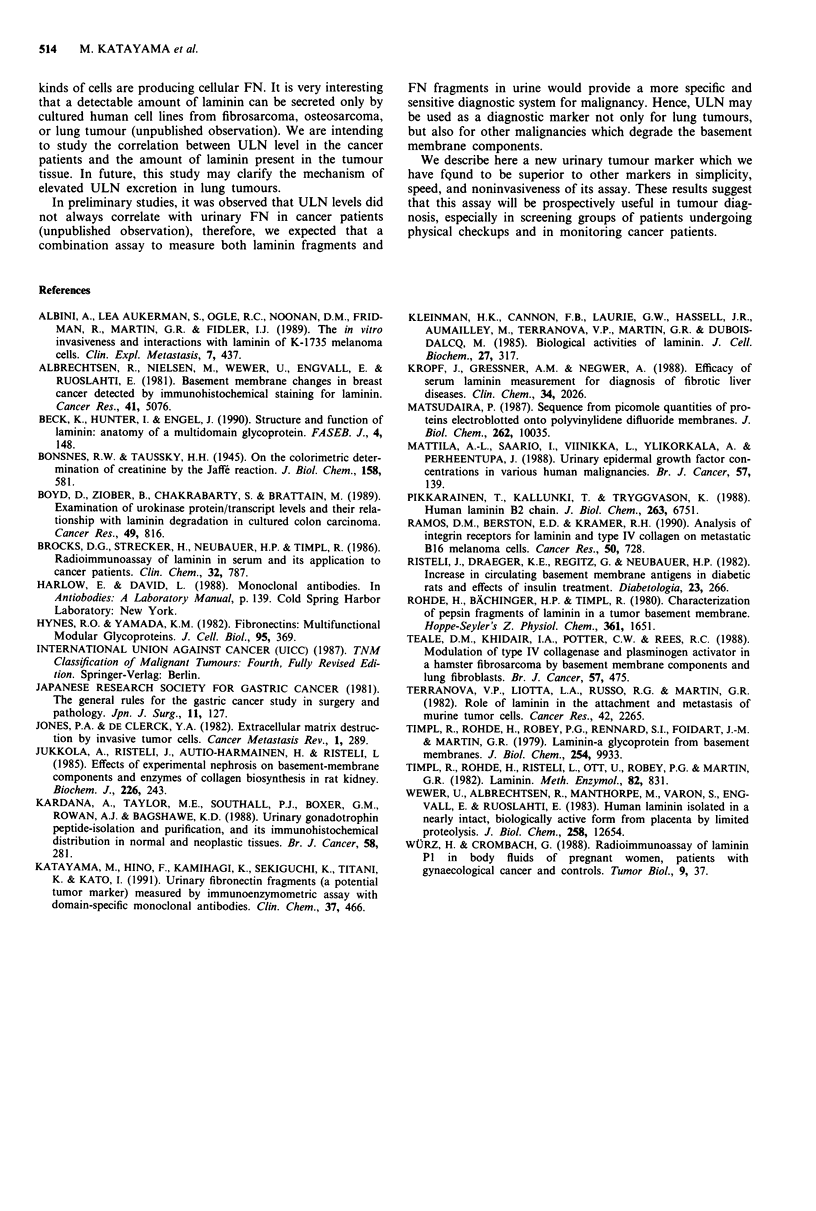# Urinary laminin fragments as a tumour marker potentially reflecting basement membrane destruction.

**DOI:** 10.1038/bjc.1992.105

**Published:** 1992-04

**Authors:** M. Katayama, K. Kamihagi, S. Hirai, T. Kurome, K. Murakami, F. Hino, I. Kato

**Affiliations:** Biotechnology Research Laboratory, Takara Shuzo Co. Ltd., Shiga, Japan.

## Abstract

**Images:**


*Br. J. Cancer* (1992), **65**, 509–514 © Macmillan Press Ltd., 1992

### Urinary laminin fragments as a tumour marker potentially reflecting basement membrane destruction

M. Katayama, K. Kamihagi, S. Hirai, T. Kurome, K. Murakami, F. Hino & I. Kato

*Biotechnology Research Laboratory, Takara Shuzo Co. Ltd., Seta 3-4-1, Otsu, Shiga 520-21, Japan.*

**Summary** The presence of soluble laminin fragments in urine of healthy subjects, patients with diabetes, and patients with tumours was studied using sandwich immunoenzymometric assay technique. The form of urinary laminin (ULN) fragments was dramatically different from that of intact laminin, so ULN could be detected only by using monoclonal antibodies. Mean levels of ULN in lung tumour were significantly higher (171 $\mu$g gram$^{-1}$ creatinine) than those in healthy subjects, patients with diabetes, patients with stomach tumour, and patients with colon tumour (respectively 91, 92, 77 and 53 $\mu$g gram$^{-1}$ creatinine). Immunopurified ULN fragments showed an apparent molecular mass of 42 KD on electrophoresis. This fragment was recognised as being derived from the N-terminal region of laminin B2 chain, because the N-terminal residues of ULN were found to be completely homologous to B2 chain. These data suggested that ULN was almost all fragmented, consisted mainly of N-terminal domain of the B2 chain, and was suspected of a tumour-associated protein fragments probably derived from basement membrane degraded proteolytically by tumour cells. ULN, increased in tumour patients, could be a potential clinical marker for monitoring the turnover of basement membrane in tumours.

Laminin, a large multidomain glycoprotein of the extracellular matrix, has attracted much interest because of its importance in the development and maintenance of cellular organisation (Timpl *et al.*, 1979; Beck *et al.*, 1990). Important cellular functions attributed to laminin include stimulation of growth and neurite outgrowth promotion (Kleinman *et al.*, 1985). Recently it has been shown that laminin is synthesised by various tumour cells (Albrechtsen *et al.*, 1981). It has also been found that laminin interacts preferentially with malignant tumour cells via the specific laminin receptors exposed on the cell surface and enhances the metastatic phenotype and cell-surface protease activity (Albini *et al.*, 1989; Ramos *et al.*, 1990; Terranova *et al.*, 1982; Teale *et al.*, 1988).

A soluble form of laminin, present in serum and other body fluids, has been measured for monitoring patients with several disorders (Kropf *et al.*, 1988; Risteli *et al.*, 1982; Brocks *et al.*, 1986; Würz & Crombach, 1988). Some clinical reports observed a significant elevation of serum laminin levels in various tumour patients, and speculated on the possible causes of the increase of laminin in serum as being increased laminin synthesis or increased proteolytic degradation by the tumour cells (Brocks *et al.*, 1986). It was also reported that serum laminin consisted of intact molecules or P1 fragments. Such P1 fragments are most resistant to proteases, and consist of around 300 KD central domain of the cross molecule with no terminal globular domains (Beck *et al.*, 1990; Brocks *et al.*, 1986). The presence of laminin in rat urine was first reported in 1985 (Jukkola *et al.*, 1985), and measurements of human urinary laminin (ULN) in pregnancy using a commercial radioimmunoassay (RIA) have been performed (Würz & Crombach, 1988). However, the structural analysis of human ULN antigens and measurements of ULN levels in several patients have not yet been performed.

The present study was undertaken to describe the relation between ULN levels and cancer stage, and to assess the possible diagnostic value of ULN fragments in lung tumours. We also describe how target ULN molecules were found to be apparently about 42 KD fragments, degraded products derived from N-terminal regions of the laminin B2 chain.

Correspondence: M. Katayama.
Received 3 July 1991; and in revised form 11 November 1991.

### Materials and methods

### *Monoclonal antibodies*

Human laminin, of nearly intact form, was purified from fresh human placenta according to a previous method (Wewer *et al.*, 1983) with the following modifications. Briefly, placenta was completely washed with phosphate buffered saline (PBS) to remove residual blood; it was homogenised in 4 M NaCl, and laminin was extracted from the insoluble residue with 0.5 M NaCl in 0.05 M Tris-HCl buffer, pH 7.0. The extract was fractionated on a column of Bio-Gel A 1.5m (BioRad Laboratories). Laminin eluted in the void volume was further purified on a Mono-Q ion exchange column by a fast protein liquid chromatography system (Pharmacia). Then, purified laminin migrated as a single molecule under non-reducing condition on 2% sodium dodecyl sulfate-polyacrylamide gel electrophoresis (SDS-PAGE) containing 0.5% agarose. Human fibrinogen (Sigma Chemical Co.) and mouse tumour laminin (Boehringer Mannheim GmbH) were used as molecular markers on electrophoresis. Anti-laminin monoclonal antibodies (MoAbs) were produced by the standard hybridoma technology (Harlow & David, 1988). Purified human laminin was used as an immunogen, antigen for screening hybridomas, and immunoenzymometric assay (IEMA) standard. Three cloned hybridomas that secreted anti-laminin MoAbs (HLN5, HLN41, and HLN82) were established. Each MoAb immobilised on agarose column could bind human laminin specifically in placenta extracts, so all of these MoAbs were recognised to be specific to human laminin.

### *Immunoassay procedure*

IEMA for laminin using two different MoAbs was constructed and performed as follows: Antibody HLN5 or HLN41 was purified by protein A chromatography and labelled with horseradish peroxidase (Boehringer Mannheim GmbH). Ninety-six-well microplates, coated with HLN82 MoAb at the antibody concentration of 10 $\mu$g ml$^{-1}$, were blocked using PBS containing 1% bovine serum albumin (BSA). One hundred $\mu$l of a standard laminin (0, 10, 20, 40, 80, 160, or 320 ng ml$^{-1}$) or a sample was added to each well. The plate was incubated for 1 h at room temperature and washed with PBS containing 0.1% Tween-20 (Wako Pure Chemical Inc.). Then, 100 $\mu$l of a solution of HLN5 or HLN41 MoAb labelled with peroxidase was added to each well, followed by incubation for 1 h at room temperature. Finally, 100 µl of 5.5 mM o-phenylenediamine dihydrochloride (Sigma Chemical Co.) soution was added as substrate and the mixture was left for 15 min at room temperature, after which the enzyme reaction was stopped by adding 100 µl of 1 M sulphuric acid. The absorbance at 492 nm was measured in a Titertek Multiscan (Flow Laboratories). Serum laminin P1 levels were determined by a commercially available RIA (Behring-Hoechst) as described elsewhere in detail (Kropf *et al.*, 1988; Brocks *et al.*, 1986). Serum samples for laminin IEMA were prediluted 4-fold with PBS containing 1% BSA.

Student's *t*-test as well as the Mann and Whitney non-parametric test were used in the analysis of ULN levels in the control and patient populations. However, since both Student's *t* and Man-Whitney tests resulted in identical *P* values, data were expressed as one *P* value. Differences were considered as significant below $P = 0.05$.

The molecular size of laminin antigens in a 1.8 ml serum sample of the patient with lung tumour, whose serum laminin level was extremely elevated, was determined by two different IEMA formats and commercial RIA after molecular-sieve chromatography on a 1.5 × 130 cm Ultrogel AcA44 (IBF biotechnics) column, equilibrated with PBS. Further clinical data of this patient with a lung tumour could not be obtained.

### *Assay of creatinine in urine*

A commercially available kit (Wako Pure Chemical Inc.) based on the method of Jaffé (Bonsnes & Taussky, 1945) was used according to the manufacturer's instructions to assay creatinine in urine. Urinary creatinine determination was performed simultaneously with IEMA for ULN, and the amount of ULN was expressed per milligram of creatinine. Results of ULN levels are given as mean ± s.d.

### *Clinicl specimens*

For evaluation of ULN assay, we collected a total of 297 spot urine samples from healthy subjects ($n = 84$), patients with diabetes mellitus (48), patients with stomach cancer (74), patients with colon cancer (10), and patients with lung cancer (81), which were diagnosed based on several clinical examinations. All clinical examinations were performed before sample collections. All the patients with diabetes used in this study were untreated and without angiopathy. All of 165 patients with pathologically and histologically proven stomach, colon, and lung cancers, who had no previous treatment of chemotherapy and radiotherapy, were entered into this study. Urine samples of tumour patients were collected before surgery.

In the additional clinical study, to evaluate the correlation between excretion level of ULN and tumour stage, we performed a detailed staging in 62 of 74 patients with stomach cancer and in 27 of 81 patients with lung cancer who provided urine samples. These 89 cancer patients were staged at the time of diagnosis after the urine samples were collected. Histological classification of stomach tumour was based on the General Rules for the Gastric Cancer Study in Surgery and Pathology in Japan (Japanese Research Society for Gastric Cancer, 1981). Stage of the lung tumour was classified according to the TNM classification of UICC (UICC, 1987). Sixty-two patients with stomach cancer were classified into seven patients with stage I, eight patients with stage II, eight patients with stage III, 27 patients with stage IV, and 12 patients with recurrent tumour. Twenty-seven patients with lung cancer were classified into four patients with stage I, six patients with stage II, four patients with stage III, nine patients with stage IV, and four patients with recurrent tumour. Metastases to distant sites were detected in one patient with stage IV stomach cancer, two patients with recurrent stomach cancer, and three patients with recurrent lung tumours. Serum and urine samples from 74 patients with stomach cancer and ten patients with colon cancer was collected simultaneously, and laminin levels of these samples were determined by using IEMA for laminin. One patient with colon cancer and one patient with stomach cancer, whose serum laminin levels were slightly and extremely elevated respectively, were selected from these 84 tumour patients. We measured laminin levels in a dilution series of these two serum samples.

For evaluation of the correlation among values of two IEMAs and commerical RIA, serum samples were collected from 42 donors, whose clincal information could not be obtained. The urine and serum samples were frozen without preservatives at − 20°C until the analysis was performed.

### *Immunoblotting analysis*

ULN was purified from seven liters of pooled urine sample from several healthy subjects using HLN82 MoAb immobilised on an agarose gel column. Bound material was eluted with PBS containing 8 M urea and dialysed against PBS. ULN was separated by 12.5% SDS-PAGE and then transferred to an Immobilon-P polyvinylidine difluoride membrane (Millipore Co.) electrophoretically. All samples were heated at 100°C for 5 min without reduction. The bound antigens were immunostained with peroxidase-labelled HLN5 MoAb and 4-chloro-1-naphthol substrate (Sigma Chemical Co.). Another transferred antigen was also stained with Coomassie Blue dye and cut out for amino-terminal amino acid sequencing with using an Applied Biosystems model 470 sequencer on the Edman degradation method according to a previous report (Matsudaira, 1987).

### Results

Purified placental laminin has the molecular mass of about 650 KD (Figure 1, lane A), and seemed to migrate slightly lower than mouse laminin with the molecular mass of 800 KD (Figure 1, lane B) and higher than human fibrinogen with the molecular mass of 330 KD (Figure 1, lane C). Immunoaffinity chromatographical analysis using immobilised MoAbs resulted in the corroborating antigenic specificity of them for laminin. This laminin used for antigen proved to be so highly purified that all MoAbs were identified to be laminin-specific, and were found not to react with some other contaminants in human placenta extracts.

In this study, we newly constructed two IEMAs based on immobilised HLN82 MoAb. The 82-5 IEMA was performed using labelled HLN5 MoAb, and the 82-41 IEMA was performed with labelled HLN41 MoAb. The precision of these two assays was evaluated by the assay of two samples of normal serum ten times each in a continuous series (intra-assay) or twice each time in ten consecutive assays (interassay). The assessment of intra-assay precision in the 82-5 IEMA and 82-41 IEMA gave CVs of 1.8% and 4.5% for a concentration of about 60 ng ml$^{-1}$, 6.4% and 6.9% for a concentration of about 145.0 ng ml$^{-1}$, respectively. Then, the assessment of interassay precision in the 82-5 IEMA and 82-41 IEMA gave CVs of 6.2% and 8.1% for a concentration of about 60 ng ml$^{-1}$, 7.4% and 8.7% for a concentration of 145 ng ml$^{-1}$, respectively. The standard curves and the dilution curves for serum samples from healthy subject, patient with colon tumour, and patient with stomach tumour in the 82-5 IEMA are shown in Figure 2. The curves appear to be parallel, indicating that the same immunoreactive substances was measured in the different dilution series. A very similar result was obtained in the 82-41 IEMA using dilution series of the same serum samples, and also in the 82-5 IEMA using dilution series of several urine samples. We examined the correlation between serum laminin levels for RIA and serum levels for IEMA. Relatively positive correlations could be found between values for RIA and values for the 82-5 IEMA ($R = 0.68$), and between values for RIA and values for the 82-41 IEMA ($R = 0.67$). The 82-5 IEMA and the 82-41 IEMA showed almost the same levels of serum laminin, and these values were correlated extremely well ($R = 0.91$). We established the molecular size of immunoreactive laminin in serum by molecular-sieve chromatograpahy (Figure 3), and

**Figure 1** Analysis of human placental laminin. Isolated human placental laminin used for immunogen (lane A), mouse tumour laminin (lane B), and human fibrinogen (lane C) were electrophoresed simultaneously in a 2% SDS-PAGE containing 0.5% agarose without reduction. Molecular weight of mouse laminin (800 KD) and human fibrinogen (330 KD) are indicated on the right.

**Figure 2** IEMA for laminin. Immobilised HLN82 MoAb and labelled HLN5 MoAb was used in this IEMA format. Standard curve (●) for laminin and dilution curves of serum samples from healthy subject (△), patients with stomach tumour (□), and patients with colon tumour (○) are shown. Serum samples were originally diluted 4-fold with PBS containing 1% BSA. Serial dilutions of pre-diluted sera are denoted above the curves.

**Figure 3** Characterisation of the size of laminin antigens in serum of lung tumur patients by molecular-sieve chromatography on a column of Ultrogel AcA44. Elution position of globular proteins (human fibrinogen, γ-globulin, BSA, ovalbumin, and lysozyme) of known molecular mass, which used for calibration, are indicated by arrows. Vo denotes the void volume of the column. Laminin levels of these eluted fractions for the 82-5 IEMA (—●—), the 82-41 IEMA (---○---), and RIA (—▲—) was measured to determine the elution position of serum laminin. Laminin levels of the 82-5 IEMA and the 82-41 IEMA was expressed as ng per ml, and laminin P1 levels of RIA was expressed as units per ml.

they appear to be relatively uniform in size with a molecular weight between fibrinogen and γ-globulin. No difference in the molecular weight was observed between the serum of several patients with cancer and in serum of healthy subjects (unpublished observation). In 15 urine samples selected randomly from 84 samples of healthy subjects, we attempted to measure ULN levels by RIA, the 82-5 IEMA, and the 82-41 IEMA. The precise determinations of ULN levels could be performed using the 82-5 IEMA predominantly, so we selected this format for measurements of laminin levels in the 297 urine specimens.

To eliminate the effect of variability in the rate of water excretion, urinary antigen concentrations were expressed in μg gram$^{-1}$ creatinine, as previously established (Mattila *et al.*, 1988). The results of laminin measurements in all urine samples are represented in Figure 4. Mean ULN level in stomach tumours was lower (74 ± 72 μg gram$^{-1}$ creatinine) than that in healthy subjects (91 ± 37) or that in diabetes (92 ± 62). Mean ULN level in colon tumours was also lower (53 ± 40) than that in healthy subjects or that in diabetes. Mean ULN level in lung tumours was higher significantly (171 ± 126) than that in healthy subjects ($P < 0.0001$), that in diabetes ($P < 0.001$), that in stomach tumours ($P < 0.0001$), or that in colon tumours ($P < 0.0001$). No significant difference was detected between mean ULN levels in any two groups of healthy subjects, patients with diabetes, patients with stomach cancer and patients with colon cancer. Using a cutoff point of 165 μg gram$^{-1}$ creatinine, mean + 2 s.d. of ULN levels in 84 healthy subjects, elevated levels of ULN were present in 48% of 81 patients with lung cancers.

Distribution of ULN levels in 62 patients with stomach cancer and in 27 patients with lung cancer could be classified into stages. ULN levels were high in none (0%) of the seven patients with stage I stomach cancer, none (0%) of the eight patients with stage II stomach cancer, two (25%) of the eight patients with stage III stomach cancer, two (7%) of the 27 patients with stage IV stomach cancer, and five (42%) of the 12 patients with recurrent stomach cancer. ULN levels were high in one (25%) of the four patients with stage I lung cancer, 2 (33%) of the six patients with stage II lung cancer, two (50%) of the four patients with stage III lung cancer, five (56%) of the nine patients with stage IV lung cancer, and

**Figure 4** ULN levels in healthy subjects, patients with diabetes, and patients with tumours. ULN levels were expressed in $\mu g\ gram^{-1}$ urinary creatinine. The mean value ± s.d. of each group is shown by a bar. Significant elevation of ULN levels in lung tumours was assessed from statistical analysis (Student's *t*-test or Mann-Whitney test, see text).

three (75%) of the four patients with recurrent lung cancer. ULN levels were extremely high in all of six patients with distant metastases. ULN levels in 74 stomach and ten colon cancers were not correlated with serum laminin levels in them (R = 0.03).

Immobilised HLN82 MoAb was presumed to bind ULN molecules effectively and could be used for antigen isolation on an agarose column. The target ULN molecule detected by using the 82-5 IEMA was visualised with peroxidase-labelled HLN5 MoAb, and the single immunostained band on the membrane showed that the apparent molecular mass of target ULN was about 42 KD (Figure 5). We also observed that labelled HLN41 MoAb did not immunostain any protein on this membrane. We performed N-terminal amino acid sequencing analysis of this 42 KD protein on another membrane and clearly identified that this protein had 15 residues, including two indeterminants (Met-Asp-Glu-Xaa-Thr-Asp-Glu-Gly-Gly-Arg-Pro-Gln-Arg-Xaa-Met-) on its N-terminal portion. The undefined residues of the sequences, indicated by Xaa on the results, were expected to be Cys, Ser, or His, which were in poor yields during the Edman cycle. These 15 amino acids seemed to be involved in the published N-terminal end sequences (NH2-Glu-Ala-Ala-Met-Asp-Glu-Cys-Thr-Asp-Glu-Gly-Arg-Pro-Gln-Arg-Cys-Met-; underlined part was matched with obtained sequence of ULN fragments) of mature laminin B2 chain polypeptide (Pikkarainen *et al.*, 1988).

### Discussion

This study reported the development of useful IEMA using immobilised HLN82 MoAb and labelled HLN5 MoAb, possible to determine not only serum laminin levels but also ULN levels in several patients and normal individual. Precision of IEMA was recognised to be acceptable, and good linearity of standard curve and sample dilutions was also observed in IEMA for laminin (Figure 2).

Many studies have already confirmed that clinical efficacy of measurement of serum laminin P1 fragments consisted of the central portion of the laminin cross (Kropt *et al.*, 1988; Brocks *et al.*, 1986; Würz & Crombach, 1988). However, none of these studies demonstrated that ULN levels could be determined reliably by RIA for laminin P1 fragments. Serum laminin levels in 42 specimens selected randomly measured using the 82-5 IEMA and the 82-41 IEMA were correlated extremely well, and positive correlations were also observed between commercial RIA and each of the IEMAs. The molecular mass of immunoreactive serum laminin for two IEMAs and RIA was shown to be similar in the molecular-sieve chromatographical analysis (Figure 3). Then, we recognised that serum laminin molecules detected specifically with the 82-5 IEMA and the 82-41 IEMA are all identical, and that these molecules in serum had almost the same antigenecity as laminin P1 fragments commonly measured using commercial RIA. In urine samples of healthy subjects, no significant levels of laminin were detected using the 82-41 IEMA or RIA. These data suggested that urine samples ordinarily contained little or no amount of intact laminin or P1 fragments, and that almost all of ULN fragments could be detected directly only by the 82-5 IEMA.

ULN levels in lung tumours were significantly elevated than ULN levels in healthy subjects, in diabetes, or in other tumours. In addition, ULN levels in three patients with acute pneumonia were not elevated (unpublished observation). Therefore, this 82-5 IEMA for ULN seems to have a satisfactory sensitivity for detecting lung tumours. Our results indicate substantial differences between the percentages of patients positive for ULN at stage I lung tumour and those at stage IV lung tumour, so we expected a direct correlation between stage of tumour and ULN level in tumour patients. Moreover, the ULN test would indeed be primarily advantageous for patients with resectable lung tumour (such as stage I). Similar correlation was observed between ULN level and disease stage also in the 62 patients with stomach cancer. However, positives for ULN test were not present in early stage stomach cancer, and increased ULN levels were oberved mostly in recurrent stomach cancer. These data demonstrated that whereas ULN level was a useful indicator of lung cancer stage, it was apparently elevated only in recurrent stomach cancer, so the ULN test may have no place in population screening of stomach and colon tumours. Contrary to our

**Figure 5** Immunoblot analysis of ULN fragments isolated from pooled normal urine samples using MoAbs. Crude ULN isolated using immobilised HLN82 MoAb was further separated on 12.5% SDS-PAGE and transblotted to the PVDF membrane. The membranes were stained with Coomassie Blue dye, and then immunostained with HLN5 MoAb conjugated with peroxidase.

expectation, serum laminin values in 84 cancer patients did not correlate with their ULN values. This result indicated that elevation of ULN level was not accompanied by elevation of serum laminin levels. Serum laminin and ULN, possibly secreted by distinct mechanisms, may therefore provide different information regarding malignant status in cancer patients. Some reports have already demonstrated that diabetic rats showed increased levels of serum laminin (Risteli *et al.*, 1982). However, the distribution of ULN levels in diabetes patients was the same as in healthy subjects (Figure 4).

This is the first study in which ULN fragments were immunopurified and analysed biolochemically. The 13 sequences of N-terminal 15 residues of isolated ULN were identical to that around the N-terminal end of human laminin B2 chain deduced from the nucleotide sequence, as reported previously (Pikkarainen *et al.*, 1988). The result of computer homology search using the EMBL data bank indicates that obtained sequences of ULN fragment is apparently derived from laminin, not from other unrelated protein. The apparent molecular mass of immunostained ULN antigen on the transblotted membrane was about 42 KD under non-reducing condition (Figure 5). Thus, ULN was apparently constituted of about 450 amino acids residues. These data enable us to conclude that ULN antigen basically comprises domain V and VI of laminin B2 chain, and that HLN5 and HLN82 MoAbs specifically react with these domains. HLN41 MoAb appeared to bind somewhere on intact laminin molecule, but failed to react with this ULN fragments and P1 fragment of laminin which comprises the inner region of the cross molecule. It appears therefore that HLN41 MoAb is recognising the other terminal domain of laminin molecule. The schematic representation of the domain structure of laminin and the binding epitopes of HLN5 and HLN82 MoAbs, which recognise the domain V or VI, are shown in Figure 6. The antigenic determinant recognised by HLN41 MoAb has not been well defined in this study and the location of its epitope cannot therefore be shown.

We speculated on the possible reasons why N-terminal domains of laminin B2 chain were excreted in urine samples and were increased in cancer patients, as follows: A previous report indicated that less laminin is present in basement membrane structures associated with metastatic tumours compared with non-metastatic or benign lesions (Albrechtsen *et al.*, 1981). A compromised basement membrane may be a consequence of increased turnover of such components as laminin and type IV collagen, or alternatively, it may be a result of decreased synthesis. The former possibility was considered in this study, since there is abundant evidence documenting the elevated expression of various proteases in separate tumour tissues and the proteolytic degradation of laminin in the extracellular matrix (Jones & De Clerck, 1982; Boyd *et al.*, 1989). The extracellular matrix components degraded by tumour cells were thought to be released first from primary sites, second to be circulated in blood flow, and finally to be excreted into urine through the kidney.

Computer analysis revealed that the B1 and B2 chains of laminin are highly homologous proteins which are probably the products of related genes (Pikkarainen *et al.*, 1988). However, some extra domains of the laminin B1 chain have no counterpart in B2, and the number of cysteine-rich repeats is 12, or one less than in the B1 chain. The degree of homology between the two chains is highest in the cysteine repeat-containing domains III, V, and VI, where 40% of the residue matches. On the contrary, the degree of complete matches between the globular domain IV and the B1 and B2 chain is only 28%, and then it was interesting that the B2 chain contains no cysteine residue in this region, whereas the B1 chain has five. It can be assumed that domain IV of B2 chain with no cystein residues is potentially sensitive to protease attack by tumour cells and tends to be destroyed around a primary or metastatic tumour site.

Previous reports showed that ULN in rats with experimental nephrosis could be measrued by RIA for laminin P2

**Figure 6** Stucture model of laminin and the binding sites of MoAbs used in IEMAs for ULN fragments. Globular regions in the A, B1, and B2 chain are indicated by patched circles. Designations of domains by roman numerals is according to a previous report (Beck *et al.*, 1990). ULN fragment is interpreted as domain V and VI in B2 chain, and the epitopes of HLN5 MoAb and HLN82 MoAb are recognised to exist within these regions.

fragments, which are suggested to contain the terminal portion of laminin molecules (Jukkola *et al.*, 1985). Laminin P2 fragments, isolated from limited-pepsin digestions of placenta or intact laminin, were reported to have a molecular mass of about 45 KD (Rohde *et al.*, 1980). ULN fragments characterised in this study, which basically consisted of N-terminal domain of the B2 chain, may have similar antigenicity to P2 fragments.

Many urinary tumour markers have been investigated previously (Kardana *et al.*, 1988; Mattila *et al.*, 1988; Katayama *et al.*, 1991). Fibronectin (FN), major extracellular matrix components with a high molecular mass, is a very popular cell adhesive glycoprotein as well as laminin and collagens (Hynes & Yamada, 1982). Recently we and our collaborators demonstrated that urinary FN fragments also significantly increase in almost all kinds of tumours including stomach, lung, liver, colon, and others (Katayama *et al.*, 1991). When extracellular matrix containing the cell adhesive proteins is degraded around tumours, these proteins may be fragmented by proteases secreted from tumours. However, the distribution of FN in tissue and body fluid was reported to be slightly different from that of laminin (Beck *et al.*, 1990). Previous immunofluorescence studies have suggested that laminin is an abundant component of basement membrane and ultrastructural studies have localised laminin to the lamina rara of epidermal and glomerular basement membranes (Timpl *et al.*, 1982). Plasma concentration of FN, reported to be 300 $\mu g\ ml^{-1}$, is significantly different from that of laminin which was reported to be about 30 $ng\ ml^{-1}$ (Hynes *et al.*, 1982; Risteli *et al.*, 1982). The elevation of urinary FN levels in various kinds of tumours appears to result from widespread distribution of FN in tissue and body fluid. On the contrary, increased ULN levels are possible to be observed in specified kinds of tumours, because laminin may be localised mostly in tissues and organs containing large amount of basement membrane. We have shown that ULN levels in lung tumours were significantly elevated compared to those in the other tumours. Increased ULN detected only in lung tumours is, however, difficult to explain. It is well known that extracellular matrix components, laminin or FN, secreted by several cultured human cells may be involved in cell-binding to these adhesive matrix proteins. Various kinds of cells are producing cellular FN. It is very interesting that a detectable amount of laminin can be secreted only by cultured human cell lines from fibrosarcoma, osteosarcoma, or lung tumour (unpublished observation). We are intending to study the correlation between ULN level in the cancer patients and the amount of laminin present in the tumour tissue. In future, this study may clarify the mechanism of elevated ULN excretion in lung tumours.

In preliminary studies, it was observed that ULN levels did not always correlate with urinary FN in cancer patients (unpublished observation), therefore, we expected that a combination assay to measure both laminin fragments and FN fragments in urine would provide a more specific and sensitive diagnostic system for malignancy. Hence, ULN may be used as a diagnostic marker not only for lung tumours, but also for other malignancies which degrade the basement membrane components.

We describe here a new urinary tumour marker which we have found to be superior to other markers in simplicity, speed, and noninvasiveness of its assay. These results suggest that this assay will be prospectively useful in tumour diagnosis, especially in screening groups of patients undergoing physical checkups and in monitoring cancer patients.

### References

ALBINI, A., LEA AUKERMAN, S., OGLE, R.C., NOONAN, D.M., FRIDMAN, R., MARTIN, G.R. & FIDLER, I.J. (1989). The *in vitro* invasiveness and interactions with laminin of K-1735 melanoma cells. *Clin. Expl. Metastasis*, **7,** 437.

ALBRECHTSEN, R., NIELSEN, M., WEWER, U., ENGVALL, E. & RUOSLAHTI, E. (1981). Basement membrane changes in breast cancer detected by immunohistochemical staining for laminin. *Cancer Res.*, **41,** 5076.

BECK, K., HUNTER, I. & ENGEL, J. (1990). Structure and function of laminin: anatomy of a multidomain glycoprotein. *FASEB. J.*, **4,** 148.

BONSNES, R.W. & TAUSSKY, H.H. (1945). On the colorimetric determination of creatinine by the Jaffé reaction. *J. Biol. Chem.*, **158,** 581.

BOYD, D., ZIOBER, B., CHAKRABARTY, S. & BRATTAIN, M. (1989). Examination of urokinase protein/transcript levels and their relationship with laminin degradation in cultured colon carcinoma. *Cancer Res.*, **49,** 816.

BROCKS, D.G., STRECKER, H., NEUBAUER, H.P. & TIMPL, R. (1986). Radioimmunoassay of laminin in serum and its application to cancer patients. *Clin. Chem.*, **32,** 787.

HARLOW, E. & DAVID, L. (1988). Monoclonal antibodies. In *Antiobodies: A Laboratory Manual*, p. 139. Cold Spring Harbor Laboratory: New York.

HYNES, R.O. & YAMADA, K.M. (1982). Fibronectins: Multifunctional Modular Glycoproteins. *J. Cell. Biol.*, **95,** 369.

INTERNATIONAL UNION AGAINST CANCER (UICC) (1987). *TNM Classification of Malignant Tumours: Fourth, Fully Revised Edition*. Springer-Verlag: Berlin.

JAPANESE RESEARCH SOCIETY FOR GASTRIC CANCER (1981). The general rules for the gastric cancer study in surgery and pathology. *Jpn. J. Surg.*, **11,** 127.

JONES, P.A. & DE CLERCK, Y.A. (1982). Extracellular matrix destruction by invasive tumor cells. *Cancer Metastasis Rev.*, **1,** 289.

JUKKOLA, A., RISTELI, J., AUTIO-HARMAINEN, H. & RISTELI, L (1985). Effects of experimental nephrosis on basement-membrane components and enzymes of collagen biosynthesis in rat kidney. *Biochem. J.*, **226,** 243.

KARDANA, A., TAYLOR, M.E., SOUTHALL, P.J., BOXER, G.M., ROWAN, A.J. & BAGSHAWE, K.D. (1988). Urinary gonadotrophin peptide-isolation and purification, and its immunohistochemical distribution in normal and neoplastic tissues. *Br. J. Cancer*, **58,** 281.

KATAYAMA, M., HINO, F., KAMIHAGI, K., SEKIGUCHI, K., TITANI, K. & KATO, I. (1991). Urinary fibronectin fragments (a potential tumor marker) measured by immunoenzymometric assay with domain-specific monoclonal antibodies. *Clin. Chem.*, **37,** 466.

KLEINMAN, H.K., CANNON, F.B., LAURIE, G.W., HASSELL, J.R., AUMAILLEY, M., TERRANOVA, V.P., MARTIN, G.R. & DUBOIS-DALCQ, M. (1985). Biological activities of laminin. *J. Cell. Biochem.*, **27,** 317.

KROPF, J., GRESSNER, A.M. & NEGWER, A. (1988). Efficacy of serum laminin measurement for diagnosis of fibrotic liver diseases. *Clin. Chem.*, **34,** 2026.

MATSUDAIRA, P. (1987). Sequence from picomole quantities of proteins electroblotted onto polyvinylidene difluoride membranes. *J. Biol. Chem.*, **262,** 10035.

MATTILA, A.-L., SAARIO, I., VIINIKKA, L., YLIKORKALA, A. & PERHEENTUPA, J. (1988). Urinary epidermal growth factor concentrations in various human malignancies. *Br. J. Cancer*, **57,** 139.

PIKKARAINEN, T., KALLUNKI, T. & TRYGGVASON, K. (1988). Human laminin B2 chain. *J. Biol. Chem.*, **263,** 6751.

RAMOS, D.M., BERSTON, E.D. & KRAMER, R.H. (1990). Analysis of integrin receptors for laminin and type IV collagen on metastatic B16 melanoma cells. *Cancer Res.*, **50,** 728.

RISTELI, J., DRAEGER, K.E., REGITZ, G. & NEUBAUER, H.P. (1982). Increase in circulating basement membrane antigens in diabetic rats and effects of insulin treatment. *Diabetologia*, **23,** 266.

ROHDE, H., BÄCHINGER, H.P. & TIMPL, R. (1980). Characterization of pepsin fragments of laminin in a tumor basement membrane. *Hoppe-Seyler's Z. Physiol. Chem.*, **361,** 1651.

TEALE, D.M., KHIDAIR, I.A., POTTER, C.W. & REES, R.C. (1988). Modulation of type IV collagenase and plasminogen activator in a hamster fibrosarcoma by basement membrane components and lung fibroblasts. *Br. J. Cancer*, **57,** 475.

TERRANOVA, V.P., LIOTTA, L.A., RUSSO, R.G. & MARTIN, G.R. (1982). Role of laminin in the attachment and metastasis of murine tumor cells. *Cancer Res.*, 42, 2265.

TIMPL, R., ROHDE, H., ROBEY, P.G., RENNARD, S.I., FOIDART, J.-M. & MARTIN, G.R. (1979). Laminin-a glycoprotein from basement membranes. *J. Biol. Chem.*, **254,** 9933.

TIMPL, R., ROHDE, H., RISTELI, L., OTT, U., ROBEY, P.G. & MARTIN, G.R. (1982). Laminin. *Meth. Enzymol.*, **82,** 831.

WEWER, U., ALBRECHTSEN, R., MANTHORPE, M., VARON, S., ENGVALL, E. & RUOSLAHTI, E. (1983). Human laminin isolated in a nearly intact, biologically active form from placenta by limited proteolysis. *J. Biol. Chem.*, **258,** 12654.

WÜRZ, H. & CROMBACH, G. (1988). Radioimmunoassay of laminin P1 in body fluids of pregnant women, patients with gynaecological cancer and controls. *Tumor Biol.*, **9,** 37.